# Supplementary material for: The Medical Triazole Voriconazole Can Select for Tandem Repeat Variations in Azole-Resistant Aspergillus Fumigatus Harboring TR34/L98H Via Asexual Reproduction
Source: J Fungi (Basel). 2020 Nov 11;6(4):277. doi: 10.3390/jof6040277 (PMC7711461; doi:10.3390/jof6040277)
Supplement: Supplementary file 1 [file jof-06-00277-s001.pdf]

Supplemental documents

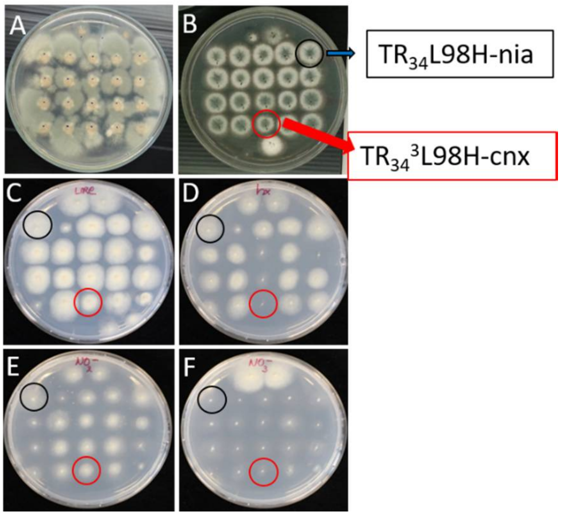

Figure S1. Isolation and classification of nitrate non-utilising mutants based on resistance to chlorate. For TR_{34}/L98H strain a *nia* (chlorate resistant, nitrate non-utilising, hypoxanthine and nitrite utilising) and for TR_{34}^{3}/L98H a *cnx* (chlorate resistant, nitrate and hypoxanthine non-utilising, nitrite utilising) mutant was selected.

A: Chlorate resistant mutants appear as sectors after UV radiation 60s (20 erg/mm^2 /sec).

B: Master plate of putative chlorate resistant mutants (from top side)

C-F: Growth test of colonies from the masterplate transferred to plates with respectively as single N-source: Urea, Hypoxathine, Nitrite and Nitrate (photograph from back side).

Table S1，The characteristics of chlorate resistant nia and cnx mutants for their ability to utilise urea, hypoxanthine, nitrite and nitrate as sole N-source.

	**N-source**			
	Ure	Nitrite	Nitrate	Hypoxanthine
TR_{34}L98H-nia	+*	+	-*	+
TR_{34}^{3}L98H-cnx	+	+	-	-

+: growth, -no growth

Table S2. The SNPs calling between the ancestor TR_{34}/L98H (V30-40) and evolved isolate TR_{34}^{3}/L98H. From left to right columns indicate **chromosome number** and **position** on reference genome (clinical strain AF293), **variant type** (ref allele / alternative allele), **genotype** calling (0=similar to reference, 1=alternative, in brackets: read depth of reference allele / read depth of alternative allele) for ancestor and **genotype** calling of evolved isolate (0=similar to reference, 1=alternative, in brackets: read depth of reference allele / read depth of alternative allele), **effect of variant** and amino acid substitution, **gene** and **function** of gene

Chromosome number	Position	Variant type (change in nucleotide)	Genotype Clinical ancestor TR_{34}/L98H (V30-40)	Genotype Evolved isolate TR_{34}^{3}/L98H	Effect of variant	Gene / Function
1	1971284-1971287	deletion<br>TTCT / -	0 (31/0)	**1 (4/23)**	Glu314<br>Frameshift	AFUA_1G06920<br>Serine / threonine protein kinase/<br>may involve in growth
2	692713	Snp<br>A / G	0 (51/0)	**1 (0/56)**	Non-synonymous<br>Leu 147 Pro	AFUA_2G02690<br>AtrR transcription factor/ plays an essential role in regulating *cyp51A* expression
4	1560507	Snp<br>C / T	0 (43/ 0)	**1 (0/38)**	Non-synonymous<br>Leu 636 Phe	AFUA_5G06450<br>Vacuolar protein sorting protein DigA/ may involve in vacuole organization

Table S3. In vitro azole susceptibility profiles of TR_{34}/L98H, Transient TR_{34}/L98H and TR_{34}^{3}/L98H

		MIC		
**Isolate code**	**Type**	**ITR**	**VOR**	**POS**
**V30-40**	Ancestor TR_{34}/L98H(V30-40)	>16	4	0,5
**V284-36**	Transient TR_{34}/L98H	>16	>16	>8
**V284-37**	TR_{34}^{3}/L98H	>16	>16	>8

A difference in resistance to VOR and POS between Transient TR_{34}/L98H and its successor TR_{34}^{3}/L98H cannot be detected in a MIC assay because both still grew at the highest concentrations of VOR and POS that can be applied.

Expression of *cyp51A* in the TR_{34}^{3}/L98H isolate, was compared with that of the TR_{34}/L98H ancestor, Transient TR_{34}/L98H and a WT control isolate. Transient TR_{34}/L98H harboured two SNPs: the AtrR mutation and another in protein PtaB (Table S4). The level of *cyp51A*-expression of the Transient TR_{34}/L98H isolate was higher than that of the ancestor, but lower than that of TR_{34}^{3}/L98H (Figure S1). These data suggest that the AtrR gene mutation alone could not cause the same expression level of *cyp51A* as TR_{34}^{3}/L98H. Therefore, in the TR_{34}^{3}/L98H, the 12-fold overexpression of *cyp51A* is likely the combined effect of the AtrR mutation and three copies of 34bp.

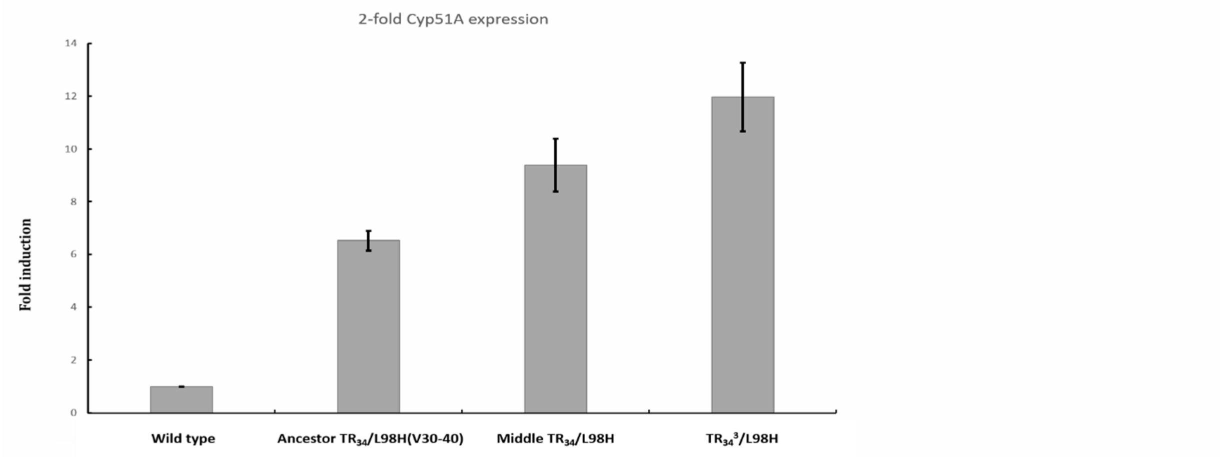

Figure S2. Expression of *cyp51A* in the TR_{34}^{3}/L98H isolate, compared with ancestry isolates TR_{34}/L98H and Transient TR_{34}/L98H, and a WT control isolate.

Table S4:The gene mutations in TR_{34}^{3}/L98H and Transient TR_{34}/L98H isolate compared with the TR_{34}/L98H (V30-40) ancestor.

	Mutations on gene / function compared to ancestor TR_{34}/L98H			
Transient TR_{34}/L98H		Non-synonymous AFUA_2G02690/ Leu 24 Pro<br>**AtrR** transcription factor/ plays an essential role in regulating *cyp51A* expression		Non-synonymous AFUA_1G06920 **PtaB** protein Ser 390 Phe, a lim-domain binding protein in Aspergillus fumigatus which regulates biofilm formation and conidiation through distinct pathways
Evolved TR_{34}^{3}/L98H	Non-synonymous AFUA_5G06450/ Leu 636 Phe<br>**Vacuolar protein** sorting protein DigA/ may involve in vacuole organization	Non-synonymous AFUA_2G02690/ Leu 24 Pro<br>**AtrR** transcription factor/ plays an essential role in regulating cyp51A expression	Glu 314 Frameshift, AFUA_1G06920<br>**Serine / threonine protein kinase**/<br>may involve in growth	
